# Genetic diversity and host adaptation of avian H5N1 influenza viruses during human infection

**DOI:** 10.1080/22221751.2019.1575700

**Published:** 2019-02-17

**Authors:** Matthijs R.A. Welkers, Hana A. Pawestri, Judy M. Fonville, Ondri D. Sampurno, Maarten Pater, Melle Holwerda, Alvin X. Han, Colin A. Russell, Rienk E. Jeeninga, Vivi Setiawaty, Menno D. de Jong, Dirk Eggink

**Affiliations:** a Department of Medical Microbiology, Academic Medical Center, Amsterdam, Netherlands; b National Institute of Health Research and Development, Ministry of Health, Jakarta, Indonesia; c Department of Zoology, University of Cambridge, Cambridge, UK; d Bioinformatics Institute, A*STAR, Singapore, Singapore; e Department of Medical Microbiology, PAMM, Veldhoven, Netherlands

**Keywords:** Influenza, H5N1, human adaptation, polymerase complex

## Abstract

The continuing pandemic threat posed by avian influenza A/H5N1 viruses calls for improved insights into their evolution during human infection. We performed whole genome deep sequencing of respiratory specimens from 44 H5N1-infected individuals from Indonesia and found substantial within-host viral diversity. At nearly 30% of genome positions multiple amino acids were observed within or across samples, including positions implicated in aerosol transmission between ferrets. Amino acid variants detected our cohort were often found more frequently in available H5N1 sequences of human than avian isolates. We additionally identified previously unreported amino acid variants and multiple variants that increased in proportion over time in available sequential samples. Given the importance of the polymerase complex for host adaptation, we tested 121 amino acid variants found in the PB2, PB1 and PA subunits for their effects on polymerase activity in human cells. We identified multiple single amino acid variants in all three polymerase subunits that substantially increase polymerase activity including some with effects comparable to that of the widely recognized adaption and virulence marker PB2-E627 K. These results indicate highly dynamic evolutionary processes during human H5N1 virus infection and the potential existence of previously undocumented adaptive pathways.

## Introduction

Highly pathogenic avian influenza A/H5N1 (HPAI H5N1) viruses continue to circulate among birds in several Asian and African countries, causing large scale outbreaks in poultry as well as sporadic human infections with high mortality [1]. To date, more than 850 confirmed human H5N1 infections have been reported, of which ∼60% were fatal, with most cases occurring in Indonesia, Vietnam and Egypt (December 2017, WHO). Global concerns persist that these viruses may evolve towards efficient transmission among humans and cause a potentially devastating influenza pandemic [2–4]. This concern is reinforced by research in ferrets showing that airborne transmission of H5N1 viruses requires only a limited number of genetic changes, several of which have already been observed in nature [2,5,6]. However, while ferrets are widely accepted as an appropriate animal model for influenza in humans, the mutations observed in ferret studies might not confer a similar transmissible viral phenotype in humans. In addition, multiple evolutionary pathways towards aerosol transmissibility between ferrets or humans are likely to exist making it challenging to assess the pandemic emergence risk of specific H5N1 viruses. For avian H5N1 viruses to cause a pandemic, they will likely have to evolve the capacity for efficient human-to-human transmission in the course of human infection following transmission from poultry. Hence, improved insights into the genetic variation and evolution of avian viruses during human infection are essential.

Although available human and avian genetic sequences in public databases are informative for the study of H5N1 influenza virus evolution, insights gained from these sequences are limited for several reasons. First, mutations are introduced during each replication cycle due to the error-prone influenza RNA polymerase [7]. Therefore, influenza viruses exist in an infected host as a dynamic viral population of genetically diverse variants, the composition of which is determined by selective pressures [8]. The vast majority of currently available sequences are solely based on consensus sequencing and do not provide information about potentially important minority variants present within the influenza virus population during infection. Second, most sequence data originate from virus strains isolated in cell culture and may therefore not accurately reflect virus diversity in tissues of the actual infected host due to differences in selective pressure [9–11]. Third, evolution of the viral population within a host is a dynamic and continuous process, and analysis of a single specimen in the course of infection merely reflects a snapshot of this process. To overcome these limitations, we investigated the genetic diversity and evolutionary dynamics of the viral population during the course of human HPAI H5N1 virus infection by whole genome next-generation sequence (NGS) analyses of clinical specimens from H5N1 virus-infected patients in Indonesia.

## Results

As part of the Indonesian national procedure for avian influenza case investigations, clinical specimens were collected from patients with suspected H5N1 virus infection and sent to the national reference laboratory for influenza at the National Institute of Health Research and Development in Jakarta, where they were stored at –80°C following laboratory confirmation by polymerase chain reaction (PCR)-based diagnostics [12]. From this repository, 52 respiratory tract specimens collected from 44 H5N1-infected patients between the years 2006 and 2011 were analysed, representing more than a quarter of the reported total number of human H5N1 infections in Indonesia during this period. The initial specimens of each patient were collected 4–15 days (median 9 days) after the onset of symptoms (Tables S1, S2). Following whole genome amplification of extracted viral RNA, NGS (Roche 454) and pre- and post-mapping quality control [13–15], we obtained on average 30,625 sequence reads per sample (range: 1,715–132,137), with near full genome coverage (Figure S1). To identify possible phenotypic changes, all genes were translated into protein sequences, using stringent quality controls for all nucleotides within codons [15].

Genetic characterization of the hemagglutinin (HA) and neuraminidase (NA) consensus sequences revealed that all viruses belonged to H5 subclade 2.1.3, prevalent in Indonesia (Figure S1). All viruses possessed a multibasic cleavage site in HA and the 20 amino acid deletion in the stalk of NA characteristic for HPAI H5N1 viruses circulating since the early 2000s [16,17]. Following this classification, we were interested in the diversity of amino acid substitutions at each protein position within and between specimens. Based on NGS of the initial specimens from the 44 patients, we observed a total of 5608 variants, where variants were defined as unique amino acid residues at a genomic position irrespective of the proportion at which they existed within the intra-host virus population. Thus, a variant could reflect the consensus amino acid found at a specific gene position within that sample or could refer to a so-called minority variant existing at proportions well below 50% within one sample (listed in Table S9). For 2986 of the 4191 genome positions with sequence information (71.25%) only a single unique amino acid was observed, indicating 100% conservation within our samples. However, multiple intra- and inter-host amino acid variants were observed at 1205 genomic positions (28.75%, Table S3).

**Figure 1.**
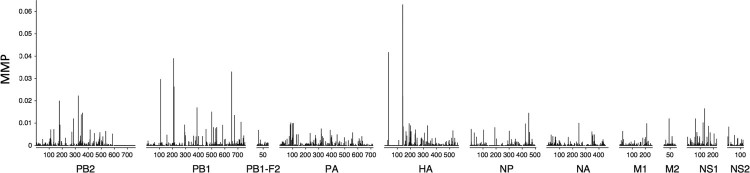
Mean minority proportion across the genome in the course of human H5N1. The mean minority proportion (MMP) is defined as the percentage of non-consensus reads per genomic position, averaged across all specimens.

To further characterize the observed within-host viral diversity, we calculated the proportion of amino acids deviating from the within-specimen consensus for each genome position and averaged this across all samples to obtain the mean minority proportion (MMP) (Figure 1). While within-host variation was observed across all genes, the highest averaged MMP values were observed in PB2, PB1, HA and NS1 and the lowest in M1, NA, NP and PB1-F2. The higher diversity in HA and genes of the polymerase complex may reflect differences in the evolutionary dynamics between genes.

To investigate the relevance of the observed viral diversity during human H5N1 virus infection for human adaptation, we initially focused on the presence of previously reported amino acid substitutions associated with airborne transmission between ferrets [5,6,18]. Virus sequence data from Indonesian patients are particularly relevant since the ferret experiments by Herfst et al. were based on the evolution of an Indonesian clade 2.1.3 reference virus [5]. Of the substitutions identified in these studies, only the PB2-E627 K and HA-N220 K changes were detected, in 1 of 17 (at 100% of the virus population) and 1 of 41 (at 6.1%) evaluable specimens respectively (Table 1). The N220 K substitution in HA (H5 numbering, corresponding to position 224 in H3 numbering) is associated with a change in receptor specificity from avian α2,3-linked to human-like α2,6-linked sialic acids [6,19], while an important role of the PB2-E627 K substitution for mammalian adaptation of influenza viruses is widely recognized [20–22]. Interestingly, at several positions implicated in airborne transmissibility between ferrets we observed minority variant populations harboring alternative amino acid residues other than those described to play a role in human adaptation (Table 1). This diversification could reflect alternative evolutionary pathways or genetic drift following relaxation of purifying selection in the absence of avian selective pressures in the human host. Including spatio-temporally matched avian samples in our analyses would have been a valuable comparison, however, such samples were not available within the study.

**Table 1.** Amino acid residues at positions implicated in airborne transmission of H5N1 viruses [5,6,18].

Position	Number of samples with information	Variants (proportion within specimens)	
PB2-E627K	17	E: 16 (81.5–100%); K: 1 (100%)	
PB1-H99Y	25	H: 25 (99.8–100%)	
HA-H103Y^a^	9	H: 9 (100%)	
HA-N154D^a^	41	N: 41 (98.5–100%)	
HA-T156A^a^	41	T: 41 (98.9–100%)	
HA-N220L^a^	41	N: 41 (93.8–100%); K: 1 (6.1)% D: 1 (3.7%)S: 1(2.8%)	
HA-Q222L^a^	41	Q: 41 (80–100%); R: 3 (1.5–20%) K: 1 (5.2%)	
HA-G224S^a^	41	G: 41 (97.4–100%); R: 1 (2.6%)	
HA-T315I^a^	39	T: 39 (71.8–100%); K: 2 (6.4–28.1%)	

^a^H5 numbering, excluding signal peptide. Corresponding to position 107, 158, 160, 224, 226, 228 and 319 In H3 numbering.

To further understand whether and to what extent the amino acid variation observed during human H5N1 infection is reflective of adaptive evolutionary processes or merely reflects natural variation of a purely avian virus, we hypothesized that truly adaptive variants should be observed more frequently in viruses isolated from humans than in those isolated from poultry.

**Figure 2.**
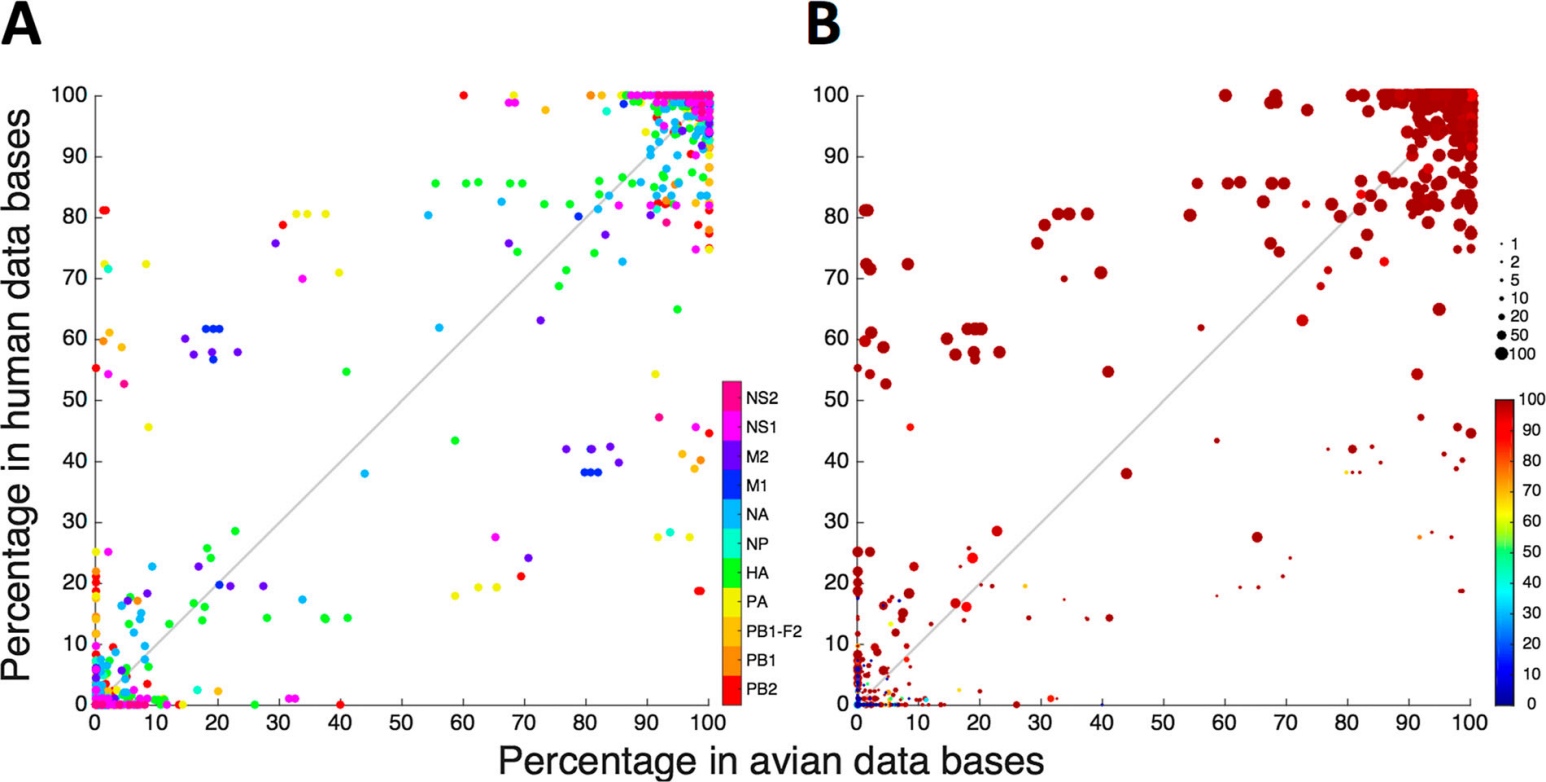
Prevalence of variants observed during human infection in publicly available sequences from avian (x-axis) and human (y-axis) H5N1 isolates from Indonesia. Each variant, defined as a unique combination of position and amino acid, is represented by a dot. (A) Prevalence of variants per viral segment. Each of the 5608 variants detected in respiratory specimens from 44 H5N1-infected patients is represented by a dot, where the colour indicates the gene in which the variant was located; (B) As (A), but displaying the percentage of samples in which the variant was detected, indicated by dot size, and the average proportion at which the variant was present within these samples, indicated by dot colour: a large red dot represents a variant that is found in many samples, at a high proportion in those samples.

To make a first comparison between the observed genetic diversity in our specimens and avian isolates from Indonesia, we calculated the root mean square deviation (RMSD). RMSD is a measure for genetic distance and visualizes the difference in amino acid distributions between our next generation sequencing data and the sequences of a set of representative chicken H5N1 isolates from Indonesia [23] (*see supplemental experimental methods*). Therefore, this analysis shows to what extent the genetic variation observed within our human H5N1 sequences, differs from genetic variation of the reference set. The use of a combination of 18 reference sequences and the determination of the mean RMSD over the 44 samples included in the study, allows the removal of less relevant minority variants detected in our NGS data. Our analyses identify a series of positions within the genome of H5N1 that differ from the selected reference viruses (Figure S3). To deepen the comparisons of variants observed in our analyses and available avian H5N1 sequences, we compared the prevalence of each amino acid variant observed in our patients in all publicly available consensus sequences of Indonesian H5N1 viruses from human and avian origin, respectively, excluding isolates originating from patients in our study (Figure 2(a)). When considering all variants, irrespective of their prevalence in our specimens or the viral proportions at which they were detected within individual specimens, the prevalence of observed variants in the human and avian H5N1 virus sequence databases was highly correlated (Pearson R: 0.99; Figure 2(a)), also when analysing genes individually (Table S5). The majority of observed variants were common in both Indonesian avian and human H5N1 virus isolates, i.e. 4147 of 5608 (74%) variants were present in more than 90% of available sequences of both avian and human origin (Figure 2(A), top right-hand corner). Moreover, these common variants were observed in the majority of our patient specimens where they existed as majority virus populations (Figure 2(b) large red symbols in top right corner). This indicates that the majority of observed variants are not specific for avian or human infection but represent the most common (consensus) amino acids at positions in the genome of human and avian H5N1 viruses circulating in Indonesia.

We next focused on the observed variants in our study that are less prevalent (<90%) in publicly available sequences. Variants that were more common in our specimens (large symbols Figure 2(b)) and existed as majority variants within individual samples (red colour), were more prevalent in publicly available sequences from human than avian Indonesian H5N1 virus isolates (*p *< 0.00001). Sensitivity analyses using varying thresholds for inclusion of variants (e.g. number of sequence reads and proportion of variants within a sample) showed similar findings (Figure S5, Table S8). While the possibility of sampling bias in publicly available sequence databases cannot be excluded, the overrepresentation of variants observed in our patients in sequences of human isolates might indicate selective advantages of these variants in the human host. This is particularly true for variants rarely or not previously reported in avian sequences but relatively common in human sequences (left upper quadrant Figure 2(b)).

**Figure 3.**
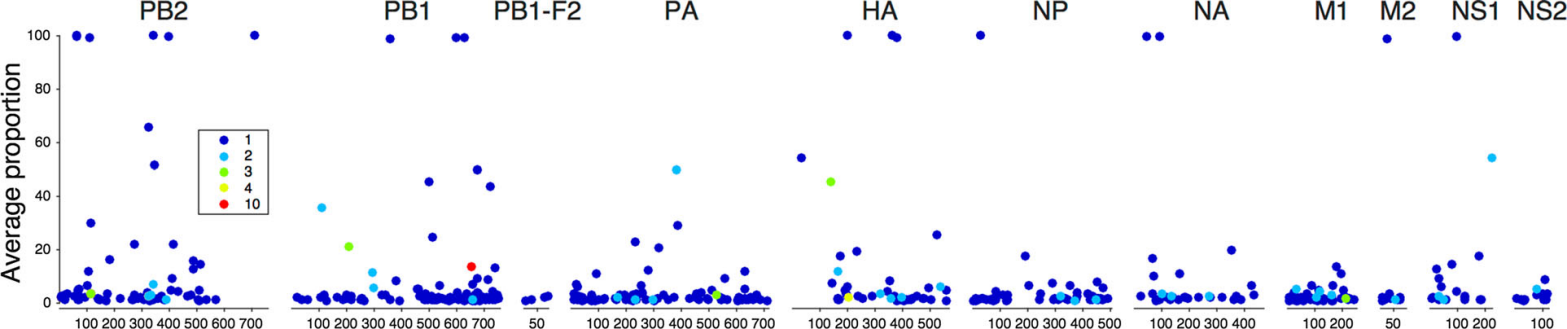
Variants not previously reported in H5N1 virus. Gene position of variants and average proportion at which these were detected within specimens are represented of 467 variants observed in patient specimens that have not been reported in publicly available sequences of avian or human H5N1 isolates. The symbol colour indicates the number of samples in which a variant was detected.

We identified 467 amino acid variants that have not been reported previously in either human or avian H5N1 virus isolates globally. These variants were observed throughout the genome and the number of variants per gene correlated strongly with the sequence lengths of the genes (Pearson R: 0.93, Table S6). While most of these variants were observed only in single specimens at low proportions, 59 were present at substantial proportions exceeding 10% of the viral population, 17 of which approached 100%. Furthermore, 38 variants were observed in multiple patients, 7 of which at proportions exceeding 10% (Figure 3, Figure S6). The absence of these variants in publicly available sequences may be explained by the fact that the latter sequences are usually generated from virus isolates obtained after *in vitro* culture in cells or embryonated eggs, which presents different selective pressures compared to human tissues [9–11]. The variants identified here might therefore potentially reveal novel adaptive pathways of the virus.

**Figure 4.**
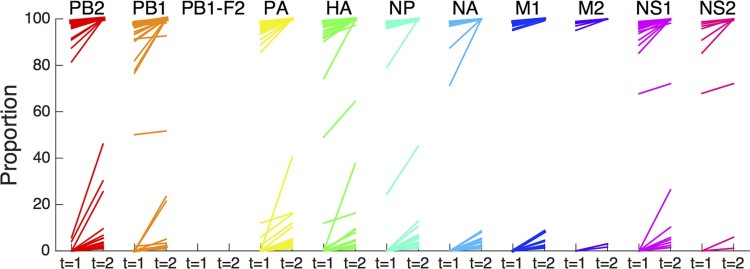
Proportional increase of variants during course of infection. Represented are 499 variants observed in sequential specimens from 8 patients, depicted per gene segment, that increased by at least 1% over 1–2 days. Initial sample was collected after a median of 8.5 days (range: 5–10) after onset of symptoms. Each line represents a single amino acid variant.

A hallmark of adaptive evolutionary processes is the selective outgrowth of variants with enhanced fitness (adaptation) during the course of infection. We analysed available serial samples collected at 1–2 day intervals from 8 patients. Only sample pairs originating from the same type of sample specimen (e.g. throat swab) were included as differences in sample location could affect virus population composition. We found that the proportions of 499 amino acid variants increased over time (Figure 4), associated with concomitant decreases in proportions of one or more other amino acids at that position (Figures S4, S7). For many of these variants, the increases in proportions were below 5% and occurred within the ranges of 1–5% or 95–100% of the viral population. However, 77 variants showed increases of more than 5%. Although more than two sequential samples are required, originating from longer time spans, to be able to properly address selection of specific substitutions, this data might suggest selective outgrowth during human infection (Table S7).

Overall, these results show high genetic diversity during human infections with HPAI H5N1. To further assess a possible functional role of the observed genetic diversity for human adaptation we performed functional assays. Because of the important role for the influenza virus polymerase complex in virulence and host adaptation and the high genetic diversity in the polymerase genes observed in our analyses, we next investigated the effect of observed mutations in the PB2, PB1 and PA genes on polymerase activity. The most widely studied avian-to-human influenza virus adaptation marker is the E627 K substitution in the virus PB2 gene [20,21,24]. Avian influenza viruses in wild aquatic birds almost exclusively contain PB2-627E, while most human influenza strains (e.g. seasonal influenza viruses H3N2 and H1N1 prior 2009) contain PB2-627 K which enhances polymerase activity and efficient replication of influenza in humans [21,24]. Unlike observations from human infections with other H5N1 clades, the lack of prevalence of PB2-E627 K virulence marker was apparent. Similarly, other reported virulence markers or adaptive substitutions within the polymerase complex were not present in our samples [25]. For phenotypic characterization of other observed substitutions, we selected a subset of variants that were preferentially observed in previously reported human isolates (top left section of Figure 2(a,b), Figure S8), those that were not previously reported in H5N1 viruses (Figures 3, S9) or those showed an increase in proportion during infection (Figure 4, Table S7). A total of 121 substitutions were selected: 63 in PB2, 39 in PB1 and 19 in PA. We next created a consensus sequence of known avian clade 2.1 H5N1 virus sequences from Indonesia to generate a suitable genetic context for testing polymerase activity. This consensus sequence was closest to the A/Indonesia/BL/2003 strain. Segments PB2, PB1, PA, and NP of this strain were cloned into expression vectors (*see supplemental experimental methods*) and substitutions were introduced using site-directed mutagenesis. For each substitution, influenza polymerase activity was measured using a minigenome reporter assay which makes use of a firefly luciferase gene containing the non-coding regions of the influenza NS gene segment [26].

**Figure 5.**
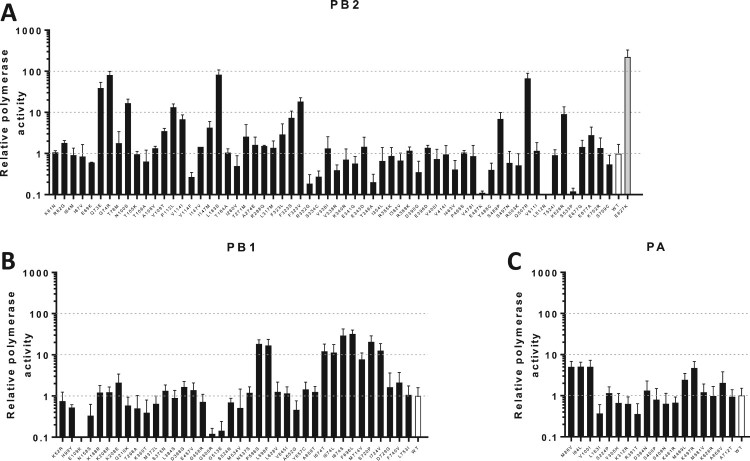
Polymerase activity of avian H5N1 influenza virus containing substitutions appearing during human infection. A mini-replicon assays was performed to screen for possible human adaptive changes within the polymerase complex/. Plasmids encoding for the PB2, PB1, PA and NP gene of avian H5N1 strain A/chicken/Indonesia/BL/2003 were co-transfected into human HEK293 T cells with a plasmid encoding for a vRNA consisting of the firefly luciferase flanked by the non-coding regions of influenza A virus NS1. Luciferase activity was measured 24 h post-transfection and incubation at 37°C. The activity of the luciferase was normalized using a control plasmid expressing the Renilla luciferase. Polymerase activity of wild type was set to one and depicted in white and human adaptive substitution PB2-E627 K is depicted in gray. Polymerase complexes containing substitutions in PB2, PB1 and PA are depicted in panels (A), (B) and (C) respectively.

Of the PB2 substitutions tested, eight showed >10 fold-increases in polymerase activity, reaching polymerase activity similar to PB2-E627 K (Figure 5(a), Table S10). Although only few PB1 substitutions are known to confer comparably dramatic effects on polymerase function [27], we identified eight substitutions that resulted in 10–40-fold increases in activity (Figure 5(b), Table S10). Substitutions in PA were not associated with >10 fold-increases, but five substitutions showed 5-7-fold increases in activity (Figure 5(c)). A small number of recent studies also used the preference of specific substitutions in human isolates to investigate possible human adaptation. However, our data identifies a large number of previously undescribed variants with major effect on polymerase activity and these studies often only identified substitutions that have relatively small effects on polymerase activity compared to our studies and/or did not consider the genetic background of the virus strain or subtype in which the substitutions were observed [26–28].

**Figure 6.**
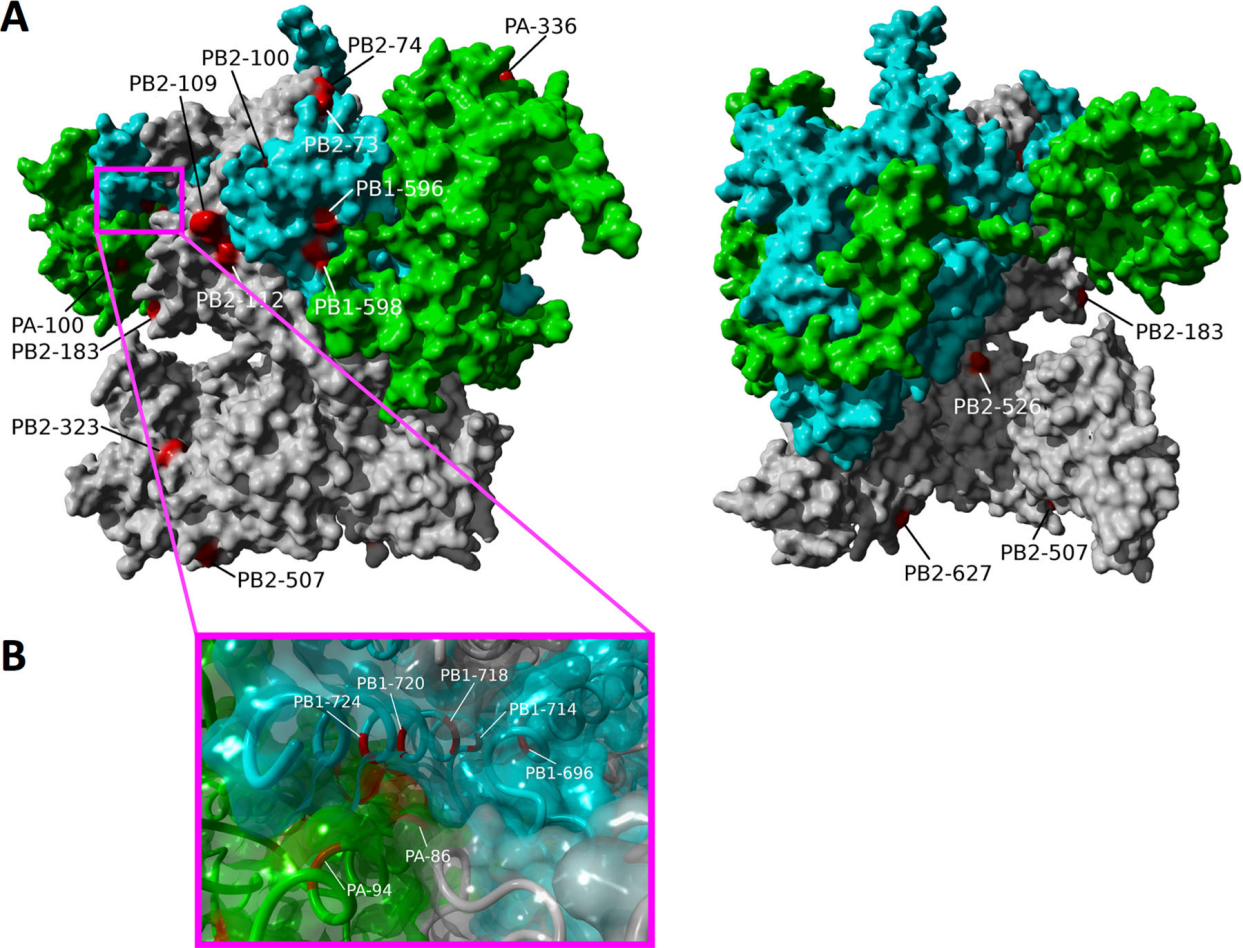
Position of polymerase complex substitutions that show large effects on polymerase activity in human cells. (A) Location of all substitutions showing an increase in polymerase activity of over 5 fold are shown of the molecular structure of the influenza A polymerase complex (PDB 4WSB). The substitutions that resulted in substantial activity increases can be divided into two groups: (1) sites on the complex surface (PB2-L183S, G74R, PB2-Q507R, PB2-F323 V, PB1-P596S, PB1-L598P, PB2-K526R, PA-L336M) and (2) sites at subunit interfaces: PB1 C-terminus and PA endonuclease domains (PB1-720, PB1-724, PA-94, PA-86) and the PB1 C-terminus and PB2 N-terminus domains (PB2-73, PB1-696, PB2-100, PB2-112, PB1-674) as shown in panel (B).

The substitutions that resulted in changes in polymerase activity were located either on the polymerase complex surface or at the interfaces between polymerase subunits. For the surface sites, such as PB2-L183S (83-fold increase in polymerase activity), PB2-G74R (81-fold), PB2-Q507R (67-fold), PB2-F323 V (18-fold), PB1-P596S (18-fold), PB1-L598P (17-fold), PB2-K526R (9-fold), and PA-L336M (7-fold) (Figure 6), the enhanced replication activity in human cells is likely attributable to interactions with host and/or viral factors. For example, PB2-E627 K is a surface site and mediates host-specific replication activity through interactions with the host cellular protein ANP32A [20]. In the case of PB2-K526R, which sits on the outer rim of the tunnel leading to the polymerase active site, residues immediately adjacent structurally (PB2-142R and 143R) have been shown to be important for viral replication and transcription [29,30]. The surface substitutions identified here are dispersed across the complex surface suggesting that host adaptation may be associated with multiple molecular mechanisms (alternative from PB2-E627 K), most of which are yet to be determined.

For the sites at subunit interfaces, the substitutions with large effects on polymerase activity were concentrated at the interfaces of the PB1 C-terminus and PA endonuclease domains and the PB1 C-terminus and PB2 N-terminus domains (Figure 6). Substitutions in the PB1 C-terminus and PA endonuclease domains include PB1-S720P (21-fold), PB1-I724 V (13-fold), PA-I94L (5-fold) and PA-M86 V (5-fold) and multiple contact points between the PB1 C-terminus and PB2 N-terminus domains include PB2-Q73E (32-39-fold), PB1-F696L (32-fold), PB2-N100S (17-fold), PB2-P112L (13-fold) and PB1-I674 T/L/S (11-30-fold). There have been few functional studies on these regions of the polymerase complex primarily because these sites form the structure that encapsulates the polymerase catalytic core and are highly conserved among publicly available avian and human influenza virus sequences. Interestingly, multiple substitutions in these domains are associated with substantial increases in polymerase activity in human cells. Due to its location, the PB1-674 likely interacts with both PB2 and the 3’ end of the vRNA promoter and the human mutations at this position are both polar relative to the isoleucine in the avian wild-type virus possibly indicating the site's role in augmenting promoter binding.

## Discussion

In summary, we investigated the within-host evolution of H5N1 viruses during human infections. Our observations show high genetic diversity during human infections with HPAI H5N1 and dynamic, potentially adaptive evolutionary processes, as suggested by an overrepresentation of observed variants in reported sequences of H5N1 viruses isolated from humans and increases in proportions of several variants over time. While known mutations reported to confer airborne transmission in ferrets were rare in our human specimens, we identified multiple substitutions within all three subunits of the influenza virus polymerase complex that substantially increase polymerase activity; in some cases, to levels similar to that of the widely recognized adaption and virulence marker PB2-E627 K. These results indicate highly dynamic evolutionary processes during human H5N1-infection and the potential occurrence of previously undocumented adaptive pathways.

## Material and methods

### Clinical samples of human influenza A/H5N1 infection

As part of the national procedure for avian influenza case investigation in Indonesia, respiratory specimens were collected from suspected H5N1 cases and sent to the national reference laboratory for influenza at the National Institute of Health Research and Development (NIHRD) in Jakarta. The NIHRD is the reference laboratory under the Indonesian Ministry of Health responsible for laboratory testing and event-based surveillance of emerging infectious diseases in humans, including avian influenza H5N1 virus. Because Indonesian clinical specimens are obtained from suspected H5N1 cases as part of the national outbreak procedure for HPAI H5N1 case investigations, requirement for informed consent has been waived by the Indonesian Ministry of Health. Samples were stored at –80°C following laboratory confirmation by polymerase chain reaction (PCR)-based diagnostics. From the specimen repositories available at NIHRD we studied 52 respiratory tract specimens from 44 patients who were diagnosed with H5N1 infection between 2006 and 2011, representing more than a quarter of the total number of 163 reported Indonesian H5N1 patients during this period. Demographic and clinical data of these patients are shown in Tables S1, S2. Specimen selection was based on a maximum cycle threshold (CT) value of 33 in the diagnostic RT–PCR to guarantee sufficient target RNA for whole genome amplification, and/or the availability of sequential samples. Sequential specimens collected at 1–2 day intervals were available from 8 patients, including serial throat swabs from 5 patients and serial nose swabs, bronchial suctions and endotracheal washes from one patient each.

### RNA extraction, cDNA synthesis and PCR amplification

For the human H5N1 samples 200 µl of clinical sample was added to 400 µl lysis-binding buffer (High pure RNA isolation kit, Roche) and RNA extraction was subsequently performed using the High Pure RNA isolation kit (Roche) with an on-column DNase treatment according to the manufacturer's protocol. Total RNA was eluted in a volume of 50 µl elution buffer (Roche) and directly used for reverse transcription and amplification. cDNA was synthesized using the Uni12M primer (AGCRAAAGCAGG) [31] and the Superscript III First-Strand Synthesis System according to the manufacturer's protocol (Invitrogen), followed by amplification of the whole genome in an overlapping amplicon approach using degenerative primer sets (primer sequences available upon request) [18,32]. PCR reactions were performed using Platinum Taq DNA Polymerase High Fidelity (Invitrogen). Thermal cycling conditions were: denaturation at 95°C for 5 min; 40 cycles of 95°C for 30 s, 50°C for 30 s, 68°C for 1 min; and final extension at 68°C for 5 min.

### Next-generation sequencing and quality control

For each sample, 5 µl of PCR product was combined and the DNA concentration of the PCR mixture was determined using the Qubit dsDNA high sensitive assay kit (Invitrogen). Samples were diluted to a DNA concentration of 50 ng/µl followed by ligation of 454 sequencing adaptors and molecular identifier (MID) tags using the SPRIworks Fragment Library System II for Roche GS FLX* DNA Sequencer (Beckman Coulter), excluding fragments smaller than 350 basepairs, according to the manufacturers protocol to allow for multiplex sequencing per region. The quantity of properly ligated fragments was determined based on the incorporation efficiency of the fluorescent primers using FLUOstar OPTIMA (BMG Labtech). Emulsion PCR, bead recovery and enrichment were performed manually according to the manufacturers protocol (Roche) and samples were sequenced in Roche FLX + 454. Standard flowgram format (sff) files containing the filter passed reads were split based on the molecular identifier (MID) sequences into separate sample-specific fastq files using the readset parser function of the QUASR package version 7.0 [13]. After removal of primer and adapter sequences by trimming the first and last 30 nucleotides of each read, an error-correction procedure was performed on all MID-split, primer-removed datasets as follows. First, nucleotides with a phred score below 28 were removed from the 3’-end of the sequence read until the first occurrence of a nucleotide with a phred score of 28 or higher. Second, a minimal median per read quality of phred 30 and minimal resulting read length of 50 nucleotides was assured using the quality control function of the QUASR version 7.0.1 (settings –m 30 –l 50). All reads were subsequently mapped to clade 2.1 reference sequence A/Indonesia/5/2005 (taxonomy ID 400788) using the Burrows–Wheeler Aligner (BWA) version 0.6.1-r104 (bwasw mapping option with default settings) [33]. Sequence alignment map (SAM)-files were parsed to remove both the unmapped and multiple mapped reads while preserving the mapped read with the highest mapping quality, generating the final cleaned datasets [34].

The amino acid variation in each sample was determined by first correcting each mapped read for insertions and deletions based on the reads’ CIGAR string and subsequent translation in three possible open reading frames (ORFs), disregarding codons that contained 1 or more nucleotides with a phred score below 20 (corresponding to an error rate of 0.1%). An amino acid overview was created for the proteins PB2, PB1, PB1-F2, PA, HA, NA, NP, M1, M2, NS1 and NS2 using the correct ORF positions. All amino acid positions covered by primers used in PCR amplification were discarded for analysis as residual (mutated) primer sequences might cause artificial sequence variation. Throughout the manuscript, the H5 numbering system is used [35].

Following amplification of extracted RNA, NGS (Roche 454) and pre- and post-mapping quality control, we obtained on average 29.005 sequence reads per sample (median 24.140, range: 1.398–132.137) of the 52 samples (including 8 sequential samples), with near full genome coverage (Figure S1).

### Threshold settings for variants

The following criteria were used for inclusion of a variant in our analyses: a minimum proportion of the variant within the sample of 1% (to mitigate the effects of amplification and sequencing errors [13,14,35,36], and a minimum of 5 sequence reads for the variant. Sensitivity analyses using different threshold settings are shown below under “Sensitivity analyses”. This data selection and all subsequent analyses were performed in Matlab R 2015 (The Mathworks) unless otherwise indicated.

### Polymerase activity (minigenome) assay

A consensus sequence was determined for al chicken isolates from Indonesia (GISAID) and the closest isolate was selected: isolate A/chicken/Indonesia/BL/2003. This virus isolate was used to further investigate polymerase activity. The open reading frame (coding region) of the PB2, PB1, PA and NP genes was cloned into the pPPI4 expression vector [37] using Gibson assembly (New England Biolabs). Mutations in PB2, PB1 and PA (Table S10) were introduced using QuikChange II Site-Directed Mutagenesis Kit (Agilent).

A plasmid coding for a model viral RNA (vRNA), consisting of the firefly luciferase (FF) open reading frame flanked by the noncoding regions of segment 8 of an H5N1 influenza A virus under the control of a human pPolI was used for minigenome assays [26]. Production of mRNA of this FF vRNA is solely possible by transcription of the produced vRNA by the influenza virus polymerase complex. Therefore, the FF activity is a measure of the amount of mRNA produced and thereby of the polymerase complex activity. Transfection of pRL (Promega) served as an internal control to normalize variation in transfection efficiency and sample processing.

HEK-293 T cells were seeded one day prior to the experiment into 96 wells plates. 25 ng of the FF reporter plasmid, 50 ng of each of the plasmids encoding PB2, PB1, and PA and 100 ng of NP and 2 ng of the Renilla luciferase expression plasmid in 50 μl Opti-MEM (Gibco, Thermo Fisher) were mixed with 50 μl Optim-mem containing Lipofectamine 2000 (Invitrogen, Thermo Fisher) in a 1:3 ratio and incubated for 20 min at room temperature. 20 μl of the transfection mixture was added to each well. Each transfection was performed in quadruple in at least two independent experiments. 24 h post transfection, luminescence was measured using the Dual-Luciferase Reporter Assay System (Promega) using a GloMax luminometer according to the manufacturer's instructions (Turner BioSystems).

### Molecular modelling

All structural inferences were made using the polymerase complex of A/little yellow-shouldered bat/Guatemala/060/2010 (H17N10, PDB: 4WSB) with YASARA, version 18.2.7 (www.yasara.org).
